# Supplementary material for: The genetic framework of shoot regeneration in Arabidopsis comprises master regulators and conditional fine-tuning factors
Source: Commun Biol. 2020 Oct 2;3:549. doi: 10.1038/s42003-020-01274-9 (PMC7532540; doi:10.1038/s42003-020-01274-9)
Supplement: Supplementary file 1 — Supplementary Information [file 42003_2020_1274_MOESM1_ESM.pdf]

Supplementary Information

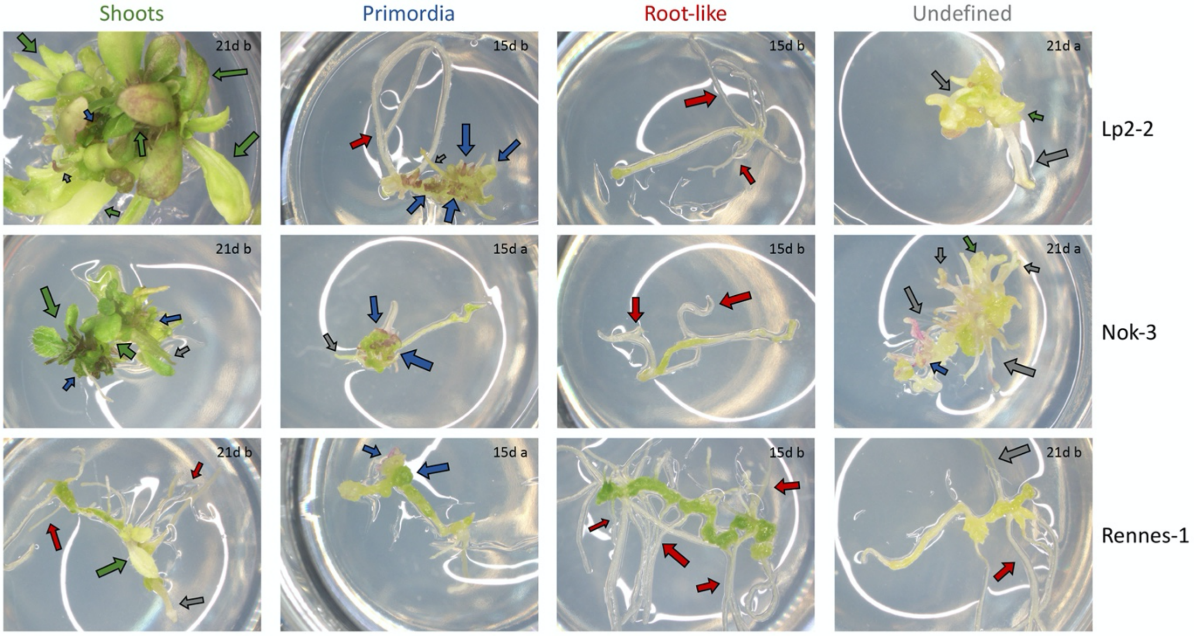

Supplementary Figure 1. Representative images of shoots, shoot primordia, root-like structures and undefined structures formed by explants of *Arabidopsis* accessions Lp2-2, Nok-3 and Rennes-1 after 15 or 21 days on SIM following protocol a or b (see individual pictures for details). Images are classified according to their most abundant feature and columns show natural variation in each phenotype. Green, blue, red and grey arrows respectively indicate shoots, shoot primordia, root-like structures and undefined structures.

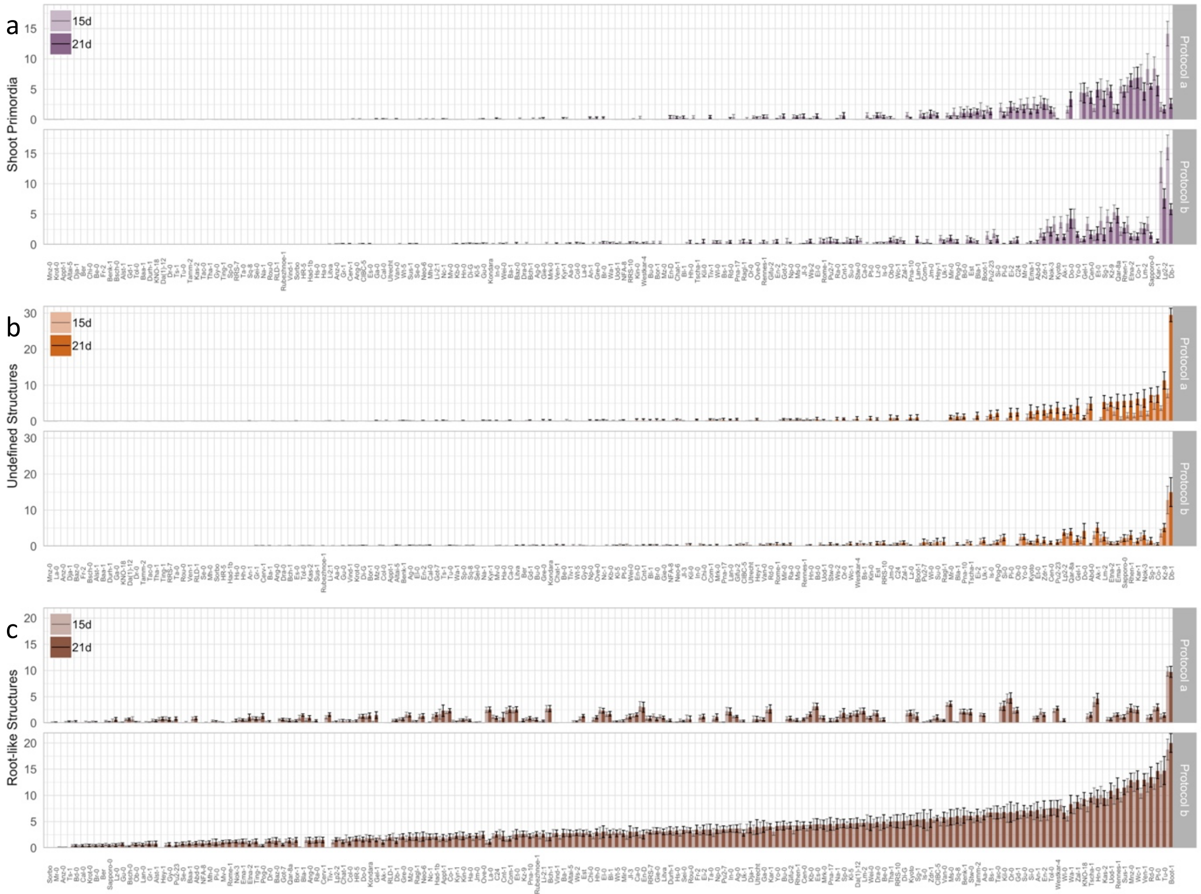

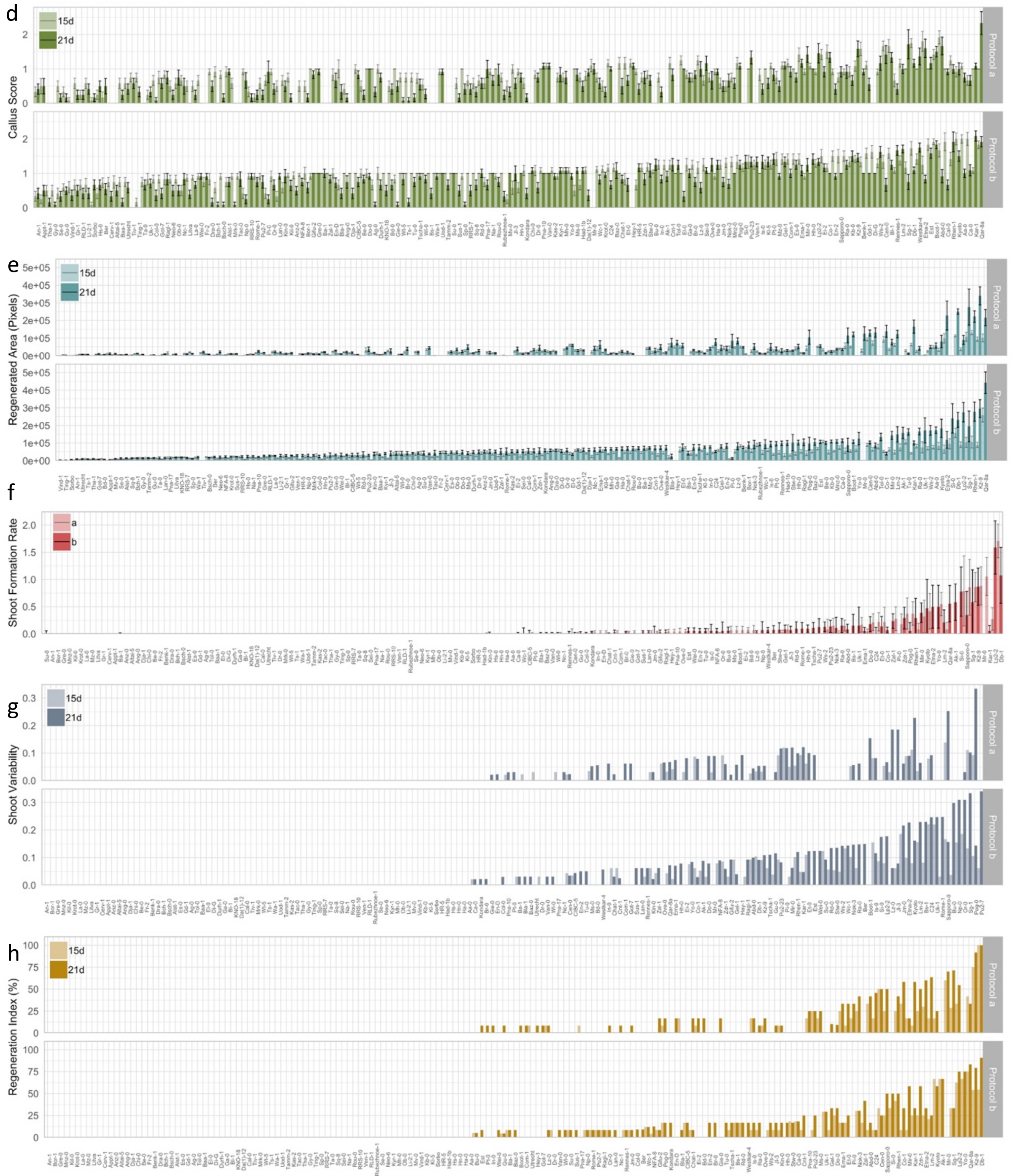

Supplementary Figure 2. Bar charts showing phenotypic variability in recorded traits other than shoot numbers, after 15 and 21 days on SIM using protocol a and b. For shoot primordia (**a**), undefined (**b**) and root-like structures (**c**), the y-axis reflects average counts, while for callus (**d**) it shows a score from 0 to 5 indicating the amount and greening of callus tissue. Regenerated area (**e**) is expressed in pixels, shoot formation rate (**f**) in shoots per day (between 15 and 21 days on SIM) and shoot variability (**g**) is calculated as the standard deviation of shoot numbers divided by the regeneration index (**h**), which is the percentage of explants forming at least one shoot. Where possible, standard errors are provided as error bars, based on n = 12 independent biological replicates.

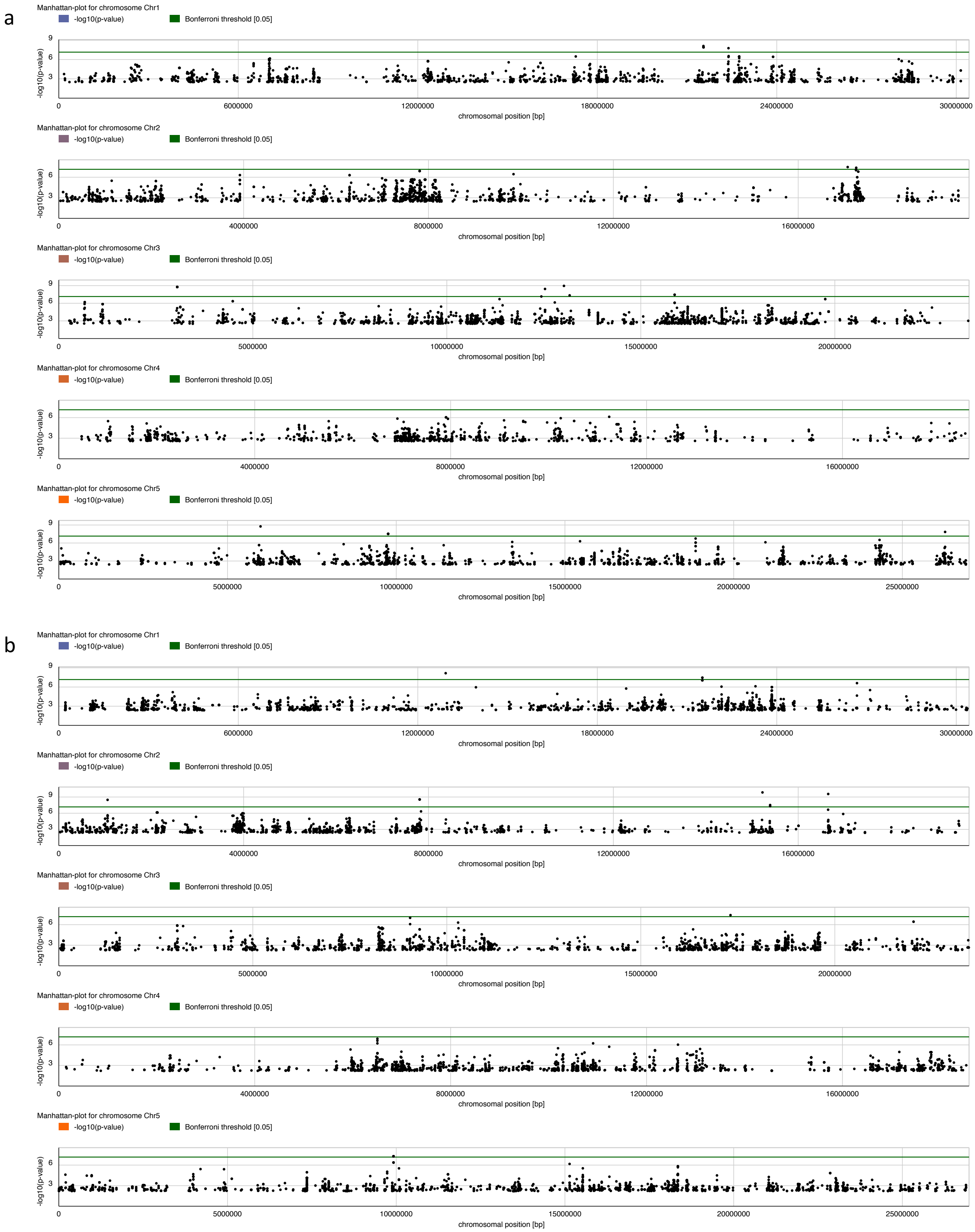

Supplementary Figure 3. Manhattan plots for all chromosomes showing SNP associations to regenerated shoot numbers after 21 days on SIM with protocol a (**a**) and b (**b**). Green lines mark a Bonferroni-corrected significance threshold of 5%, deduced from an efficient mixed-model association expedited (EMMAX) test with n = 129 and n = 149 independent samples for protocol a and b.

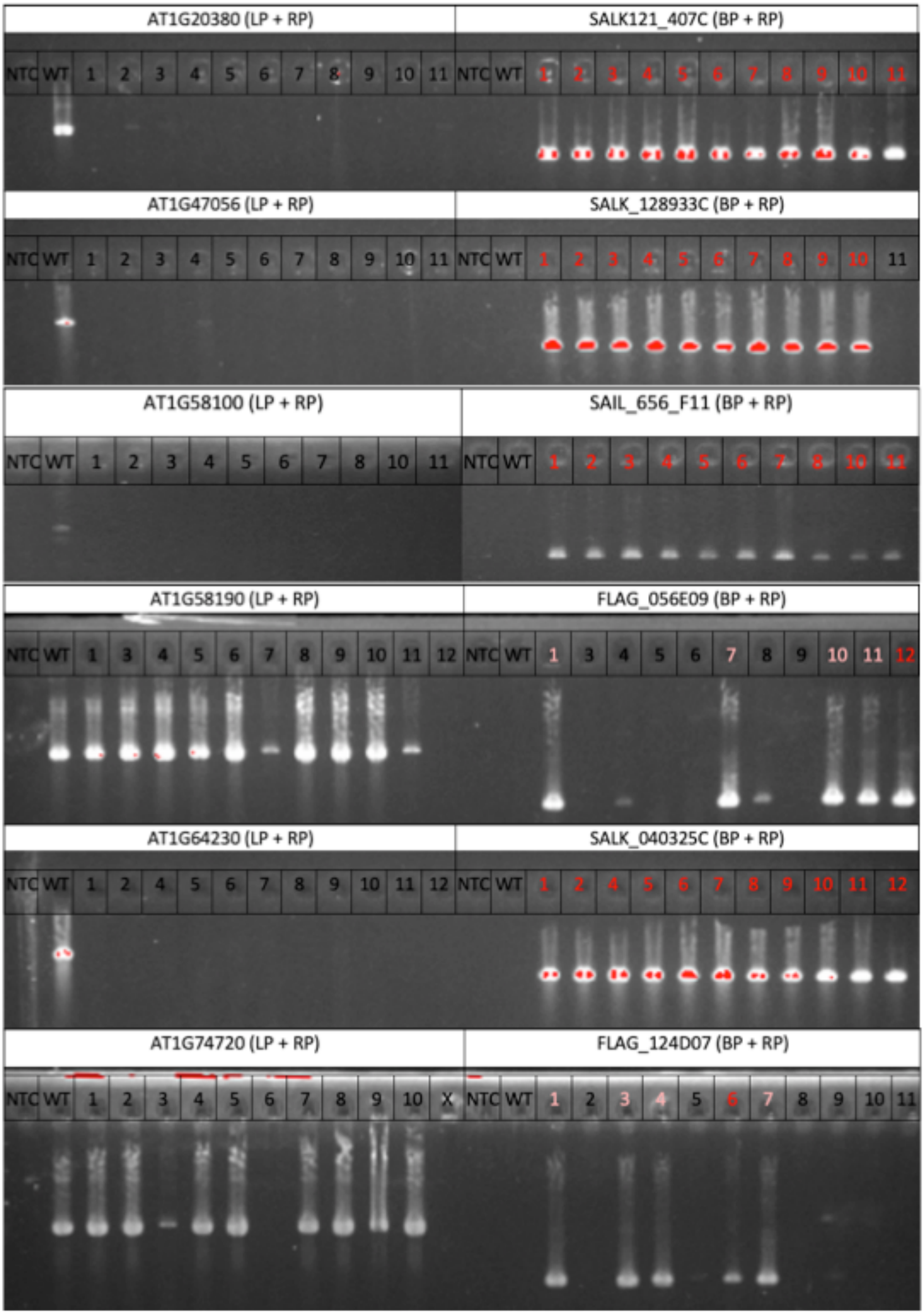
AT1G20380 (LP + RP)
SALK121_407C (BP + RP)
NTC WT 1 2 3 4 5 6 7 8 9 10 11 NTC WT 1 2 3 4 5 6 7 8 9 10 11
AT1G47056 (LP + RP)
SALK_128933C (BP + RP)
NTC WT 1 2 3 4 5 6 7 8 9 10 11 NTC WT 1 2 3 4 5 6 7 8 9 10 11
AT1G58100 (LP + RP)
SAIL_656_F11 (BP + RP)
NTC WT 1 2 3 4 5 6 7 8 10 11 NTC WT 1 2 3 4 5 6 7 8 10 11
AT1G58190 (LP + RP)
FLAG_056E09 (BP + RP)
NTC WT 1 3 4 5 6 7 8 9 10 11 12 NTC WT 1 3 4 5 6 7 8 9 10 11 12
AT1G64230 (LP + RP)
SALK_040325C (BP + RP)
NTC WT 1 2 4 5 6 7 8 9 10 11 12 NTC WT 1 2 4 5 6 7 8 9 10 11 12
AT1G74720 (LP + RP)
FLAG_124D07 (BP + RP)
NTC WT 1 2 3 4 5 6 7 8 9 10 X NTC WT 1 2 3 4 5 6 7 8 9 10 11

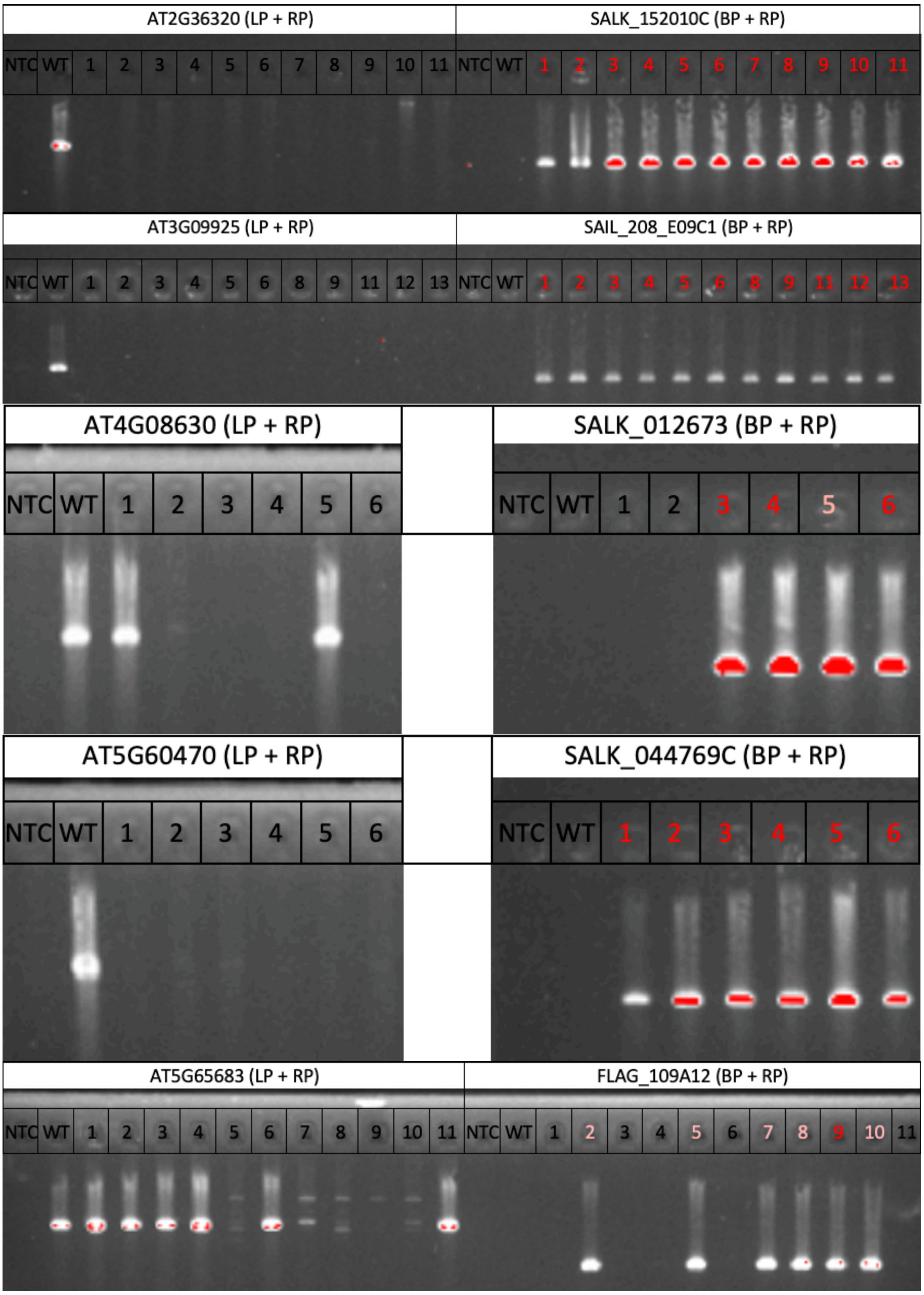

Supplementary Figure 4. PCR genotyping of T-DNA insertion lines. Gene reactions (left + right primer) are on the left and T-DNA reactions (border + right primer) are on the right. For an overview of the primers refer to Supplementary Table 1. Homozygous mutants are marked red and heterozygous inserts are pink. Numbers refer to individual plants, NTC is no-template control and WT is wild-type (either Col-0, Col-3 or WS).

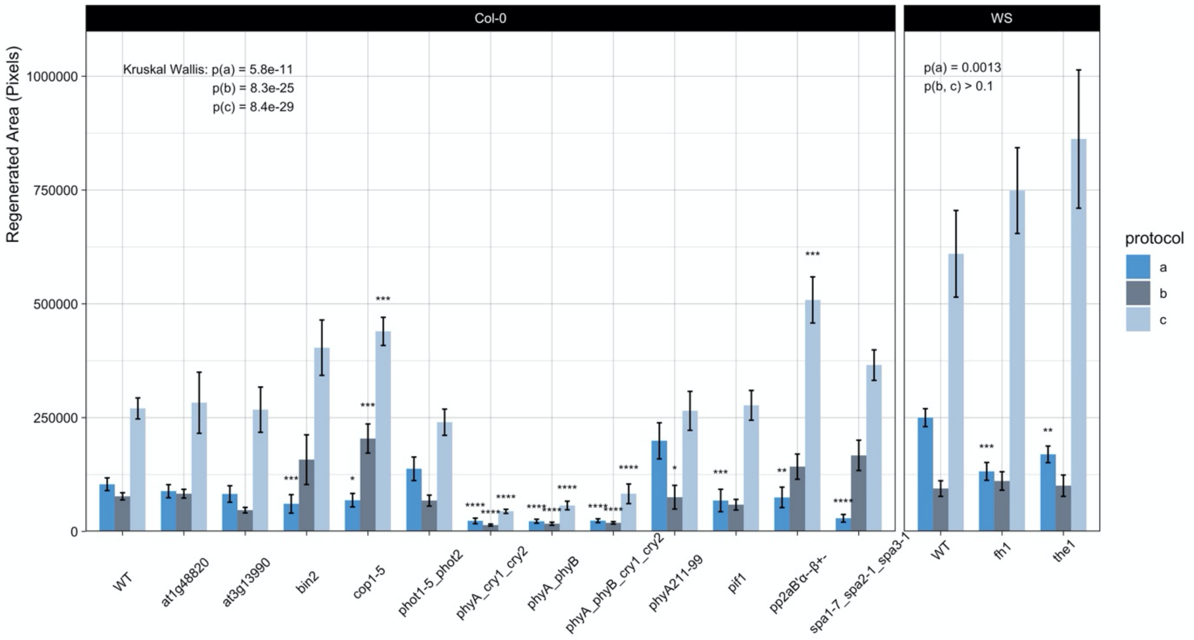

Supplementary Figure 5. Regeneration in random T-DNA insertion lines and mutants related to light signalling and phosphorylation as negative and positive controls for the data presented in Fig. 7. P-values are deduced from a global Kruskal-Wallis test and Dunn's many-to-one test for pairwise, two-sided comparisons to a single control (* = p < 0.1, ** = p < 0.05, *** = p < 0.01) and error bars reflect standard errors based on n = 24 independent biological replicates.

Supplementary Table 1. Primers used for PCR and RT-qPCR.

Gene	Primer	Sequence (5' → 3')	T (°C)
**AT1G20380 (POQR)**	SALK_121407C_LP	TTGATCAGTCCCAAGGAAATG	49
	SALK_121407C_RP	ATGTTGGTTGAGTTGACGGAG	52
**AT1G47056 (VFB1)**	SALK_128933C_LP	TGCGGTAAACCTTAAATTTTG	45
	SALK_128933C_RP	CTCGAGATTCGAGCAATGATC	50
**AT1G58100 (TCP8)**	SAIL_656_F11_LP	TGGATTGTAATGAGCTGTCCC	52
	SAIL_656_F11_RP	TTGAAGCTGATGATGAAACCC	50
**AT1G58190 (RLP9)**	FLAG_056E09_LP	GTCGAGCACTCCAAGAGATTG	53
	FLAG_056E09_RP	GGACACTTGCCAAAATTCTCTC	51
**AT1G64230 (UBC28)**	SALK_040325C_LP	GAGGTGGTAATTGCCGCTAAG	53
	SALK_040325C_RP	TTGGGTCCGTTAACAATGAAC	50
**AT1G74720 (QKY)**	FLAG_124D07_LP	CGCCATATCTTTTCATGTGTG	49
	FLAG_124D07_RP	AGACCTTCTTCACCTCTTCGC	54
**AT2G17950 (WUS)**	WUS_transcript_FW	ATCCCAGCTTCAATAACGGGAA	54
	WUS_transcript_RV	GTTTGCCCATCCTCCACCTA	55
**AT2G36320 (SAP4)**	SALK_152010C_LP	ACAAGCAAAAGCCACACAATC	51
	SALK_152010C_RP	TAGAATTTGGCACCACCAAAC	50
**AT3G09925**	SAIL_208_E09C1_LP	GTGCATGTAAATTTGCTTCCG	50
	SAIL_208_E09C1_RP	CAAACTTCAACGTGGTTGTGG	52
**AT3G54000 (TIP41L)**	TIP41L_transcript_FW	GTGAAAACTGTTGGAGAGAAGCAA	53
	TIP41L_transcript_RV	TCAACTGGATACCCTTTCGCA	54
**AT4G08630**	SALK_012673C_LP	TATATGCCGTTGTGGTAGACG	51
	SALK_012673C_RP	GTCTCATTGCTCTTCCACCAG	53
**AT4G27960 (UBC9)**	UBC9_transcript_FW	TCACAATTTCCAAGGTGCTGC	54
	UBC9_transcript_RV	TCATCTGGGTTTGGATCCGT	54
**AT5G60470 (EGRET)**	SALK_044769C_LP	ATATCCCCACTTTTCAGCCC	52
	SALK_044769C_RP	TGTATGCATGCATCTGTTTCG	50
**AT5G65683 (WAVH2)**	FLAG_109A12_LP	TTGTTTTATCGGACGGTCAAG	50
	FLAG_109A12_RP	CTCTGCAATACAAAAGGCCAC	51
**SALK T-DNA border**	SALK_LB1.3	ATTTTGCCGATTTCGGAAC	48
**SAIL T-DNA border**	SAIL_LB3	TAGCATCTGAATTTCATAACCAATCTCGATACAC	54
**FLAG T-DNA border**	FLAG_LB4	CGTGTGCCAGGTGCCCACGGAATAGT	65
