# Supplementary material for: The genetic framework of shoot regeneration in Arabidopsis comprises master regulators and conditional fine-tuning factors
Source: Commun Biol. 2020 Oct 2;3:549. doi: 10.1038/s42003-020-01274-9 (PMC7532540; doi:10.1038/s42003-020-01274-9)
Supplement: Supplementary file 2 — Description of Additional Supplementary Files [file 42003_2020_1274_MOESM2_ESM.pdf]

Description of Additional Supplementary Files

File Name: Supplementary Data 1
Description: List of all quantitative trait genes, showing significance, impact, amount and position of associated SNPs, as well as the number of phenotypes they underlie, the allele distribution between top and bottom accessions and information regarding T-DNA insertion lines, expression levels and prior knowledge.

File Name: Supplementary Data 2
Description: Results of transcriptome comparisons, containing lists of differentially expressed genes between accessions with one allele and those with the other for every candidate SNP, indicating significance, log fold changes, average expression and regression coefficients.

File Name: Supplementary Data 3
Description: Source data for Fig. 1, Fig. 3, Fig. 5, Fig. 6, Fig. 7, Supplementary Fig. 2 and Supplementary Fig. 5.

File Name: Supplementary Code
Description: Custom code for image processing (ImageJ), handling phenotypic data (R), selection of candidate genes based on association data (python), transcriptome and RT-PCR analysis (R).
