# Supplementary material for: The genetic framework of shoot regeneration in Arabidopsis comprises master regulators and conditional fine-tuning factors
Source: Commun Biol. 2020 Oct 2;3:549. doi: 10.1038/s42003-020-01274-9 (PMC7532540; doi:10.1038/s42003-020-01274-9)
Supplement: Supplementary file 7 — Reporting Summary [file 42003_2020_1274_MOESM7_ESM.pdf]

nature research

Corresponding author(s): Robin Lardon, Danny Geelen

Last updated by author(s): Aug 21, 2020

Reporting Summary

Nature Research wishes to improve the reproducibility of the work that we publish. This form provides structure for consistency and transparency in reporting. For further information on Nature Research policies, see Authors & Referees and the Editorial Policy Checklist .

Statistics

For all statistical analyses, confirm that the following items are present in the figure legend, table legend, main text, or Methods section.

n/a	Confirmed	
☐	☒	The exact sample size (*n*) for each experimental group/condition, given as a discrete number and unit of measurement
☐	☒	A statement on whether measurements were taken from distinct samples or whether the same sample was measured repeatedly
☐	☒	The statistical test(s) used AND whether they are one- or two-sided<br>*Only common tests should be described solely by name; describe more complex techniques in the Methods section.*
☐	☒	A description of all covariates tested
☐	☒	A description of any assumptions or corrections, such as tests of normality and adjustment for multiple comparisons
☐	☒	A full description of the statistical parameters including central tendency (e.g. means) or other basic estimates (e.g. regression coefficient) AND variation (e.g. standard deviation) or associated estimates of uncertainty (e.g. confidence intervals)
☐	☒	For null hypothesis testing, the test statistic (e.g. *F*, *t*, *r*) with confidence intervals, effect sizes, degrees of freedom and *P* value noted<br>*Give P values as exact values whenever suitable.*
☒	☐	For Bayesian analysis, information on the choice of priors and Markov chain Monte Carlo settings
☒	☐	For hierarchical and complex designs, identification of the appropriate level for tests and full reporting of outcomes
☐	☒	Estimates of effect sizes (e.g. Cohen's *d*, Pearson's *r*), indicating how they were calculated

Our web collection on statistics for biologists contains articles on many of the points above.

Software and code

Policy information about availability of computer code

Data collection	Images were processed using a custom ImageJ macro (version 2.0.0-rc-69/1.52j) and basic manipulation of phenotypic data was performed in R (version 3.5.2). All code is available in the Supplementary Code file (plain text).
Data analysis	Association analyses were done in easyGWAS using the EMMAX algorithm. Candidate gene selection was done using custom python code (version 2.7.15). Transcriptome comparisons and RT-qPCR analysis were performed in R (version 3.5.2). All code is available in the Supplementary Code file (plain text).

For manuscripts utilizing custom algorithms or software that are central to the research but not yet described in published literature, software must be made available to editors/reviewers. We strongly encourage code deposition in a community repository (e.g. GitHub). See the Nature Research guidelines for submitting code & software for further information.

Data

Policy information about availability of data

All manuscripts must include a data availability statement. This statement should provide the following information, where applicable:

- Accession codes, unique identifiers, or web links for publicly available datasets
- A list of figures that have associated raw data
- A description of any restrictions on data availability

The data generated and/or analysed in the current study are either included in this article as Supplementary Data or submitted to public repositories. Raw phenotype data (Fig. 1 and Supplementary Fig. 2) are available from AraPheno at https://doi.org/10.21958/study:80 and full GWAS data (Fig. 2, Fig. 5a-c and Supplementary Fig. 3) are available in easyGWAS at https://easygwas.ethz.ch/gwas/myhistory/public/24/ (accession codes for phenotypic averages underlying these results are AT1P23868, AT1P24025, AT1P24028, AT1P24031, AT1P24043, AT1P24048, AT1P24065, AT1P24068, AT1P24071, AT1P24088, AT1P25078, AT1P26148). Associations for raw phenotypes submitted to AraPheno were also recomputed using a permutation-based pipeline and published in the AraGWAS Catalog (DOI: 10.21958/gwas:1290, 10.21958/gwas:1283, 10.21958/gwas:1276, 10.21958/gwas:1269, 10.21958/gwas:1288, 10.21958/gwas:1281, 10.21958/gwas:1274, 10.21958/gwas:1267, 10.21958/gwas:1289, 10.21958/gwas:1282, 10.21958/gwas:1275, 10.21958/gwas:1268, 10.21958/gwas:1291, 10.21958/gwas:1284, 10.21958/gwas:1277, 10.21958/gwas:1270, 10.21958/gwas:1294, 10.21958/gwas:1287, 10.21958/gwas:1280, 10.21958/gwas:1273, 10.21958/gwas:1292,

10.21958/gwas:1285, 10.21958/gwas:1278, 10.21958/gwas:1271, 10.21958/gwas:1293, 10.21958/gwas:1286, 10.21958/gwas:1279, 10.21958/gwas:1272). Source data for Fig. 1, Fig. 3, Fig. 5, Fig. 6, Fig. 7, Supplementary Fig. 2 and Supplementary Fig. 5 are available in Supplementary Data 3. For questions on data availability, contact robin.lardon@ugent.be.

Field-specific reporting

Please select the one below that is the best fit for your research. If you are not sure, read the appropriate sections before making your selection.

☒ Life sciences ☐ Behavioural & social sciences ☐ Ecological, evolutionary & environmental sciences

For a reference copy of the document with all sections, see nature.com/documents/nr-reporting-summary-flat.pdf

Life sciences study design

All studies must disclose on these points even when the disclosure is negative.

Sample size	The GWAS included 190 accessions, which is based on similar studies with a successfull outcome. For phenotyping, 12 explants were analyzed per accession, T-DNA insertion mutant or chromosome substitution line. This number was determined by prior experience, feasibility and the use of 24-well plates (2 lines per plate). RT-qPCR included 3 biological replicates, concordant with the state-of-the-art.
Data exclusions	Accessions for which less than 6 data points were obtained and accessions for which no genotype data was available or whose identity was uncertain were excluded from the association analyses.
Replication	Phenotyping was performed using multiple protocols to ensure robustness and several accessions were phenotyped repeatedly, confirming that objective phenotypic values were obtained.
Randomization	Accessions were randomly divided over batches, and within each batch they were divided over plates according to a completely randomized block design. The same was done for T-DNA insertion mutants and chromosome substitution lines, which were analyzed in 1 run.
Blinding	Blinding was not relevant for this study, either because no particular outcome was expected or because it was not possible to influence the outcome during the experiment.

Reporting for specific materials, systems and methods

We require information from authors about some types of materials, experimental systems and methods used in many studies. Here, indicate whether each material, system or method listed is relevant to your study. If you are not sure if a list item applies to your research, read the appropriate section before selecting a response.

Materials & experimental systems

n/a	Involved in the study
☒	☐ Antibodies
☒	☐ Eukaryotic cell lines
☒	☐ Palaeontology
☒	☐ Animals and other organisms
☒	☐ Human research participants
☒	☐ Clinical data

Methods

n/a	Involved in the study
☒	☐ ChIP-seq
☒	☐ Flow cytometry
☒	☐ MRI-based neuroimaging
